# ReactomeGSA: new features to simplify public data reuse

**DOI:** 10.1093/bioinformatics/btae338

**Published:** 2024-05-28

**Authors:** Alexander Grentner, Eliot Ragueneau, Chuqiao Gong, Adrian Prinz, Sabina Gansberger, Inigo Oyarzun, Henning Hermjakob, Johannes Griss

**Affiliations:** Department of Dermatology, Medical University of Vienna, Vienna 1090, Austria; European Molecular Biology Laboratory, European Bioinformatics Institute (EMBL-EBI), Wellcome Genome Campus, Hinxton CB10 1SD, UK; European Molecular Biology Laboratory, European Bioinformatics Institute (EMBL-EBI), Wellcome Genome Campus, Hinxton CB10 1SD, UK; European Molecular Biology Laboratory, European Bioinformatics Institute (EMBL-EBI), Wellcome Genome Campus, Hinxton CB10 1SD, UK; Department of Dermatology, Medical University of Vienna, Vienna 1090, Austria; Department of Dermatology, Medical University of Vienna, Vienna 1090, Austria; European Molecular Biology Laboratory, European Bioinformatics Institute (EMBL-EBI), Wellcome Genome Campus, Hinxton CB10 1SD, UK; Department of Dermatology, Medical University of Vienna, Vienna 1090, Austria; European Molecular Biology Laboratory, European Bioinformatics Institute (EMBL-EBI), Wellcome Genome Campus, Hinxton CB10 1SD, UK

## Abstract

**Motivation:**

ReactomeGSA is part of the Reactome knowledgebase and one of the leading multi-omics pathway analysis platforms. ReactomeGSA provides access to quantitative pathway analysis methods supporting different ‘omics data types. Additionally, ReactomeGSA can process different datasets simultaneously, leading to a comparative pathway analysis that can also be performed across different species.

**Results:**

We present a major update to the ReactomeGSA analysis platforms that greatly simplifies the reuse and direct integration of public data. In order to increase the number of available datasets, we developed the new grein_loader Python application that can directly fetch experiments from the GREIN resource. This enabled us to support both EMBL-EBI’s Expression Atlas and GEO RNA-seq Experiments Interactive Navigator within ReactomeGSA. To further increase the visibility and simplify the reuse of public datasets, we integrated a novel search function into ReactomeGSA that enables users to search for public datasets across both supported resources. Finally, we completely re-developed ReactomeGSA’s web-frontend and R/Bioconductor package to support the new search and loading features, and greatly simplify the use of ReactomeGSA.

**Availability and implementation:**

The new ReactomeGSA web frontend is available at https://www.reactome.org/gsa with an built-in, interactive tutorial. The ReactomeGSA R package (https://bioconductor.org/packages/release/bioc/html/ReactomeGSA.html) is available through Bioconductor and shipped with detailed documentation and vignettes. The grein_loader Python application is available through the Python Package Index (pypi). The complete source code for all applications is available on GitHub at https://github.com/grisslab/grein_loader and https://github.com/reactome.

## 1 Introduction

Pathway analyses are key methods to analyze ‘omics datasets. Quantitative pathway analysis methods, such as gene set analysis, have a higher statistical power than differential expression analyses on the single-gene level (Ackermann and Strimmer 2009). The pathway level additionally directly links the observed changes to defined biological processes which greatly simplifies the interpretation of the results. Finally, when comparing datasets from different ‘omics technologies, the pathway level can function as a natural aggregator to quickly test whether the same biological changes were observed even though different proteins/genes were observed. Therefore, pathway analysis methods are important methods to analyze and compare ‘omics datasets.

The Reactome Knowledgebase is one of the leading pathway resources (Milacic *et al.* 2024). It provides access to manually curated pathways, organized as ordered networks of molecular transformations in a single consistent data model. This network is enriched with detailed annotations for each molecule and pathway. The whole data is accessible through an intuitive, interactive web-based application, where users can view the whole data at different levels of detail and get direct access to all annotations. This enables non-bioinformatic experts to quickly see the biological processes perturbed by a disease or physiological change.

In order to support quantitative pathway analyses, we recently developed the ReactomeGSA analysis platform (Griss *et al.* 2020). ReactomeGSA provides different gene set and gene set variation analysis methods on different ‘omics datasets. As a key feature, ReactomeGSA can analyze multiple datasets simultaneously, which enables users to quickly compare independent datasets on the pathway level. Recently, ReactomeGSA was found to be the best performing multi-omics pathway analysis tool in terms of validation, stability, ease of use, documentation and user-friendliness (Maghsoudi *et al.* 2022).

The continuously increasing amount of publicly available data makes it common practice for researchers to directly compare their datasets with published ones. In order to simplify this process, several resources exist that use consistent bioinformatics pipelines to re-process these datasets. This has the advantage that users can find the data in a consistent fashion, including the required meta-data. Two leading resources for these datasets are the EMBL-EBI Expression Atlas (Moreno *et al.* 2022) and the GEO RNA-seq Experiments Interactive Navigator (GREIN) (Mahi *et al.* 2019). Expression Atlas provides access to thousands of manually curated microarray, transcriptomics, and proteomics datasets. GREIN is focused on re-processing transcriptomics datasets from the Gene Expression Omnibus (GEO) and the Sequence Read Archive and contains more than 25 000 datasets at the time of writing. Unfortunately, neither of these resources provides standardized API’s that would enable external tools to directly load these datasets for further re-processing.

Here, we present a major update or ReactomeGSA where we incorporated several new features to simplify the direct access and retrieval of public datasets from Expression Atlas and GREIN. We developed a new, freely-available, open-source python package that can directly load datasets from GREIN (https://github.com/grisslab/grein_loader). ReactomeGSA additionally provides a search function that enables users to directly search for public datasets in Expression Atlas and GREIN from within ReactomeGSA, that can then be loaded into a ReactomeGSA analysis. These new features are available through a completely re-developed web interface as well as the ReactomeGSA Bioconductor R package. This major update therefore greatly simplifies the reuse and integration of public datasets in the quantitative, comparative pathway analyses provided by ReactomeGSA.

## 2 Results

**Figure 1.**
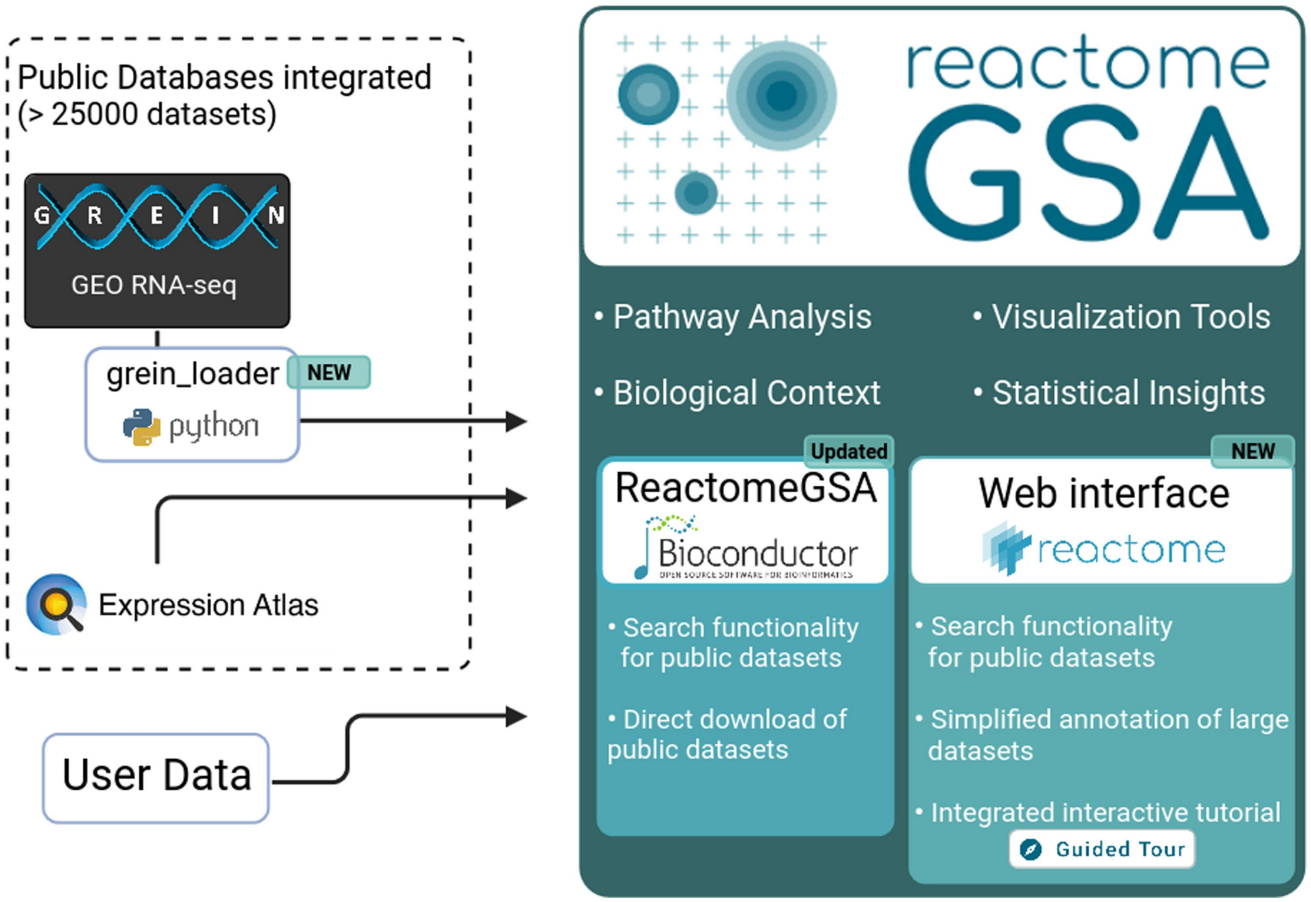
Schematic workflow of the public data integration and updated features in ReactomeGSA. Data can be directly loaded from GREIN, through the newly developed grein_loader Python package, and EMBL-EBI’s Expression Atlas. ReactomeGSA’s web interface was completely re-developed and now contains an integrated search function for all public datasets, an interactive tutorial, and improved functions to annotate large datasets. Finally, our R/Bioconductor package was updated to support the search and direct download of public datasets from all supported external resources.

ReactomeGSA provides a web-based API, available at https://gsa.reactome.org which can be accessed through two clients: our new, completely re-developed web interface, available at https://reactome.org/gsa or through the ReactomeGSA R/Bioconductor package (Fig. 1). Since both act as clients to the ReactomeGSA API, both lead to identical results. It is furthermore possible to switch between the web-based workflow and the R-based analysis and vice versa and, for example, start the analysis through the R package, while viewing the results in the interactive web-based visualization. Thereby, ReactomeGSA is catering to both expert and non-expert bioinformatics users.

### 3.1 grein_loader is a novel Python-based interface to GREIN

GREIN does not provide an API to programmatically access data. We therefore developed the novel Python package “grein_loader” (https://github.com/grisslab/grein_loader) that can directly load datasets and their associated metadata from GREIN. This replicates GREIN’s web interface and uses the underlying RShiny backend to programmatically retrieve and download the data. The data are returned as standard Python dictionaries and widely-used PANDAS data.frames respectively (McKinney 2010). Thereby, grein_loader is compatible with the majority of Python-based bioinformatics workflows. grein_loader provides two key functions. The “load_overview()” function returns the complete list of datasets currently available in GREIN. It thereby mimics the starting screen displayed by the GREIN web interface. The “load_dataset()” function takes a GEO accession id as parameter and loads the respective dataset’s metadata and expression values. grein_loader is available through pypi and can thereby be easily integrated in existing workflows.

### 3.2 Simplified public data reuse in ReactomeGSA

Simplified public data reuse was always a key goal of ReactomeGSA. Therefore, since its launch in 2020, ReactomeGSA always provided the option to directly load datasets from EMBL-EBI’s Expression Atlas resource. The process of accessing external resources is run in a dedicated kubernetes pod, which enables us to independently scale this process based on current demand. Our first version of the API requires an external dataset’s id as a minimal input in order to load it. Nevertheless, this feature had the key downside that users had to first visit the Expression Atlas website to find a dataset of interest and then copy the URL encoded dataset identifier into an input field within the ReactomeGSA web interface. Additionally, this feature was not available in the R/Bioconductor package. Overall, this led to the fact that this feature was used by only a minority of users.

We therefore significantly extended ReactomeGSA to further simplify the reuse of public data. First, we added support for GREIN through our new grein_loader package and a custom cache for GREIN datasets. Thereby, users have access to more than 40 000 additional public datasets. Similar to datasets from Expression Atlas, users can enter the respective GEO identifier and the dataset is automatically loaded into the ReactomeGSA analysis. The key advantage of this approach is that the complete metadata is loaded as well, which in our experience often is the most time consuming part of adding datasets. This new resource thereby significantly extends the amount of available public data within ReactomeGSA.

To further simplify the integration of public datasets, we extended ReactomeGSA by incorporating a keyword-based search function across all datasets available in GREIN and Expression Atlas. The new search process is implemented with the Python library Whoosh (https://whoosh.readthedocs.io/), which provides keyword based search functionalities. Whoosh requires the creation of a curated library, allowing a fast, in-memory keyword based search. In order to enable fast response, the search index is built during the weekly release process. In this process, we retrieve a complete list of all available datasets consisting of the id, title, description, and species from Expression Atlas and GREIN. This search index is then loaded into memory when the application starts to ensure fast response times. This leads to an average response time of 0.064 s per request (calculated based on *n* = 100 requests). Thereby, users can quickly find relevant public datasets and immediately integrate them into their pathway analysis.

We finally updated our ReactomeGSA R/Bioconductor package to fully support searching and loading of public datasets (from version 1.17). The ReactomeGSA R package also acts as a client to the ReactomeGSA API which ensures that both the web interface, as well as the R package produce identical results. The search results are returned in a standard R data.frame. Loaded experiments are returned as BioBase ExpressionSet objects (Huber *et al.* 2015). This workflow is therefore compatible with the vast majority of existing R packages. This feature is therefore the first integration of GREIN datasets into R/Bioconductor based workflows.

### 3.3 The re-developed ReactomeGSA web-frontend combines training with simplified data input

We completely re-developed the ReactomeGSA web-frontend to improve its usability and extend its features (Supplementary Fig. S1). The complete application was rewritten using the Angular javascript framework maintained by Google (https://angular.dev/), the UI components are based on Angular Material (https://material.angular.io/), while the state management is using NgRx (https://ngrx.io/). All of these are well established components which simplifies the long-term maintenance of this project.

As a key new feature, the user interface now offers an inbuilt, interactive tutorial. This tutorial explains all required steps to select an algorithm for data analysis, load and annotate datasets, and how to retrieve the results (Supplementary Fig. S2). This tutorial “plays” directly within the user interface. Respective controls that are relevant for the current step are highlighted with text boxes explaining the part of the workflow. Once the step is complete, the next step is displayed in the same fashion. This has the great advantage that users do not have to switch between, for example, a video, and the application.

Based on the user feedback we found that entering metadata was a major obstacle for many users. In order to improve this time consuming step, we developed a novel table component. This component supports direct copy and paste from spreadsheet applications, as well as the up- and download of the entered data. This new table component has been modularized and is kept independent from the ReactomeGSA web interface application. It is available as free and open-source software and can thereby be easily incorporated in other projects.

Finally, the new web frontend fully supports ReactomeGSA’s new search function for public datasets. As a new feature, the user interface now enables users to download both the metadata as well as the expression values of public datasets. We very much hope that this will further simplify and increase the reuse of public data.

## Supplementary Material

btae338_Supplementary_Data

## Data Availability

The grein_loader python package is freely available through the The Python Package Index (PyPi). The complete source code is available under the permissive MIT source code license on GitHub at https://github.com/grisslab/grein_loader. Reactome with all its components is available through https://reactome.org. The source code of the various components of Reactome is freely available on GitHub at https://github.com/reactome.
