# Supplementary material for: ReactomeGSA: new features to simplify public data reuse
Source: Bioinformatics. 2024 May 28;40(6):btae338. doi: 10.1093/bioinformatics/btae338 (PMC11147800; doi:10.1093/bioinformatics/btae338)
Supplement: btae338_Supplementary_Data [file btae338_supplementary_data.pdf]

ReactomeGSA: New features to simplify public data reuse - Supplementary Figures

Alexander Grentner[1], Eliot Ragueneau[2], Chuqiao Gong[2], Adrian Prinz[2], Sabina Gansberger[1], Inigo Oyarzun[1], Henning Hermjakob[2], Johannes Griss[1,2]

1 Department of Dermatology, Medical University of Vienna, Währinger Gürtel 18-20, 1090 Vienna, Austria

2 European Molecular Biology Laboratory, European Bioinformatics Institute (EMBL-EBI), Wellcome Genome Campus, Hinxton, Cambridgeshire, CB10 1SD, UK

Table of Contents

Supplementary Figure 1 **2**
Supplementary Figure 2 **3**

Supplementary Figure 1

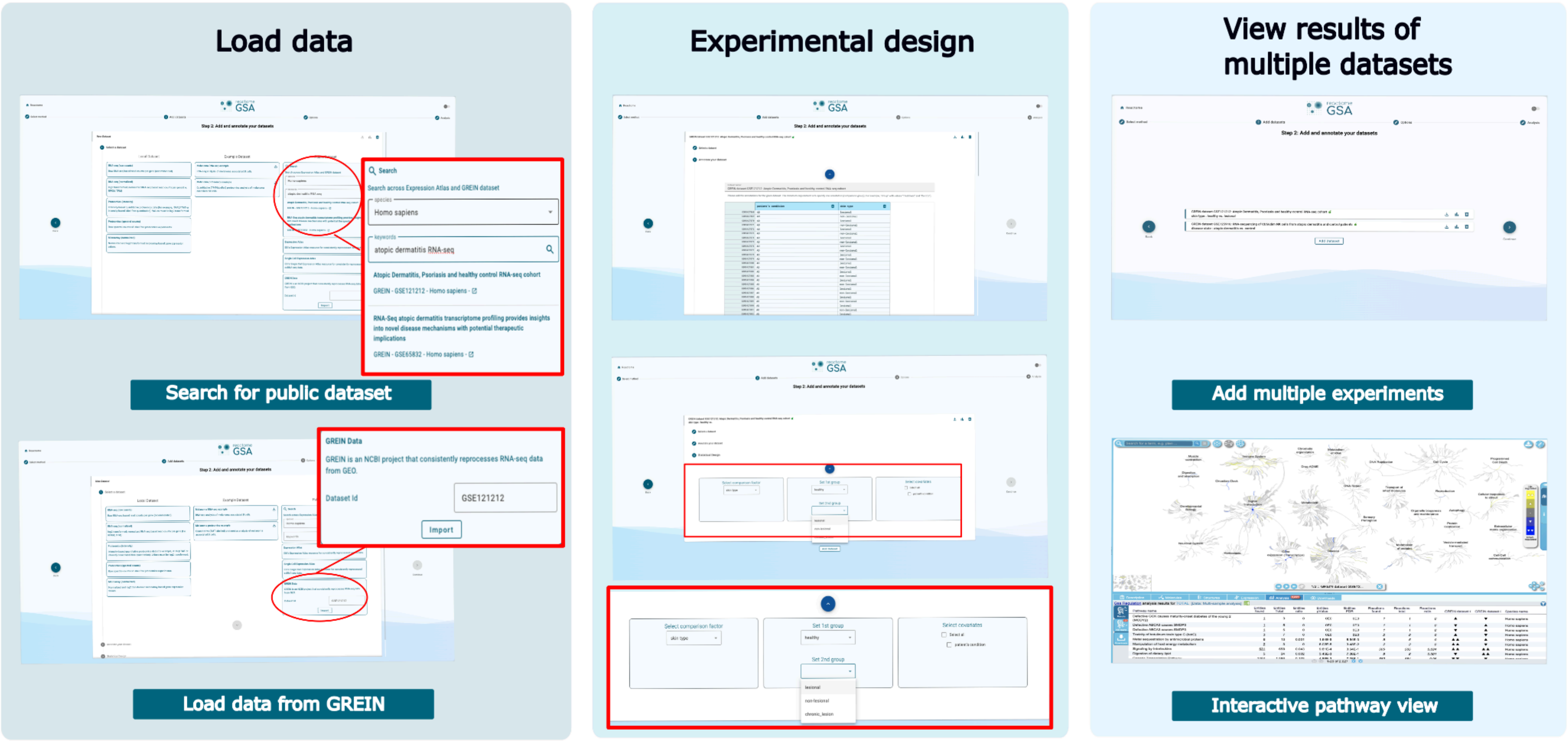

Screenshots highlighting the features of ReactomeGSA's newly developed web interface. **Load data:** The interface now provides a new function to directly search for public datasets across all supported resources (currently GREIN and EBI's ExpressionAtlas). The datasets can subsequently be directly integrated into the analysis. Additionally, datasets can also be directly loaded based on the respective identifier. **Experimental design:** The components to annotate the experimental design are based on a newly developed table component. This component supports copy & paste actions from spreadsheet applications, as well as the upload and download of the respective annotation data. Finally, the factors used for the two-group comparison can quickly be chosen based on the provided input data. **View results of multiple datasets:** ReactomeGSA supports the analysis of multiple datasets simultaneously. These are then visualized side-by-side in the interactive, web-based pathway browser.

Supplementary Figure 2

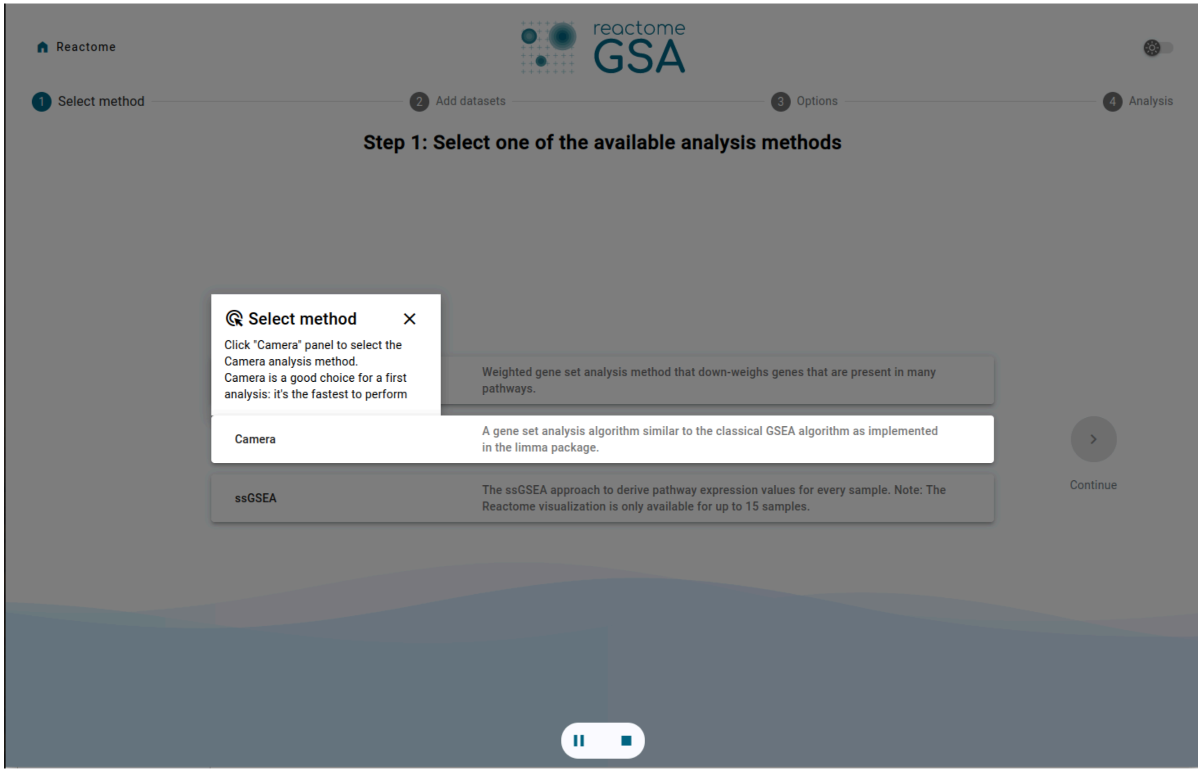

The interactive tutorial provides a step-by-step guide through ReactomeGSA use directly integrated into the web interface.
